# Supplementary figures and images for: Non-invasive skin sampling detects systemically administered drugs in humans
Source: PLoS One. 2022 Jul 26;17(7):e0271794. doi: 10.1371/journal.pone.0271794 (PMC9321436; doi:10.1371/journal.pone.0271794)

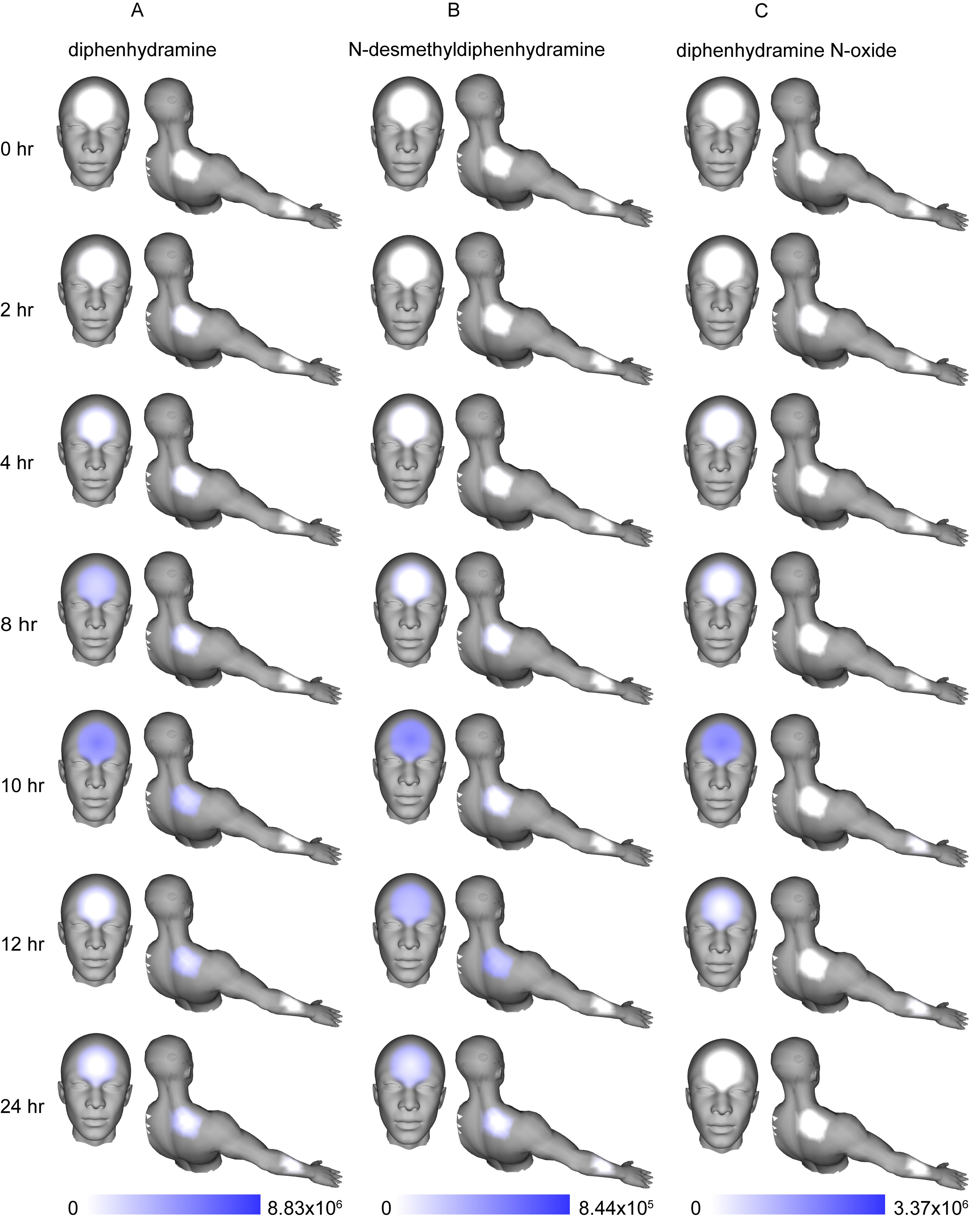

Supplement: S1 Fig — This 3D illustrative molecular map uses a white-blue color scale representative of increasing metabolite intensity for each skin site observed for each metabolite (A) diphenhydramine, (B) N-desmethyldiphenhydramine and (C) diphenhydramine N-oxide. (TIF) [file pone.0271794.s002.tif]

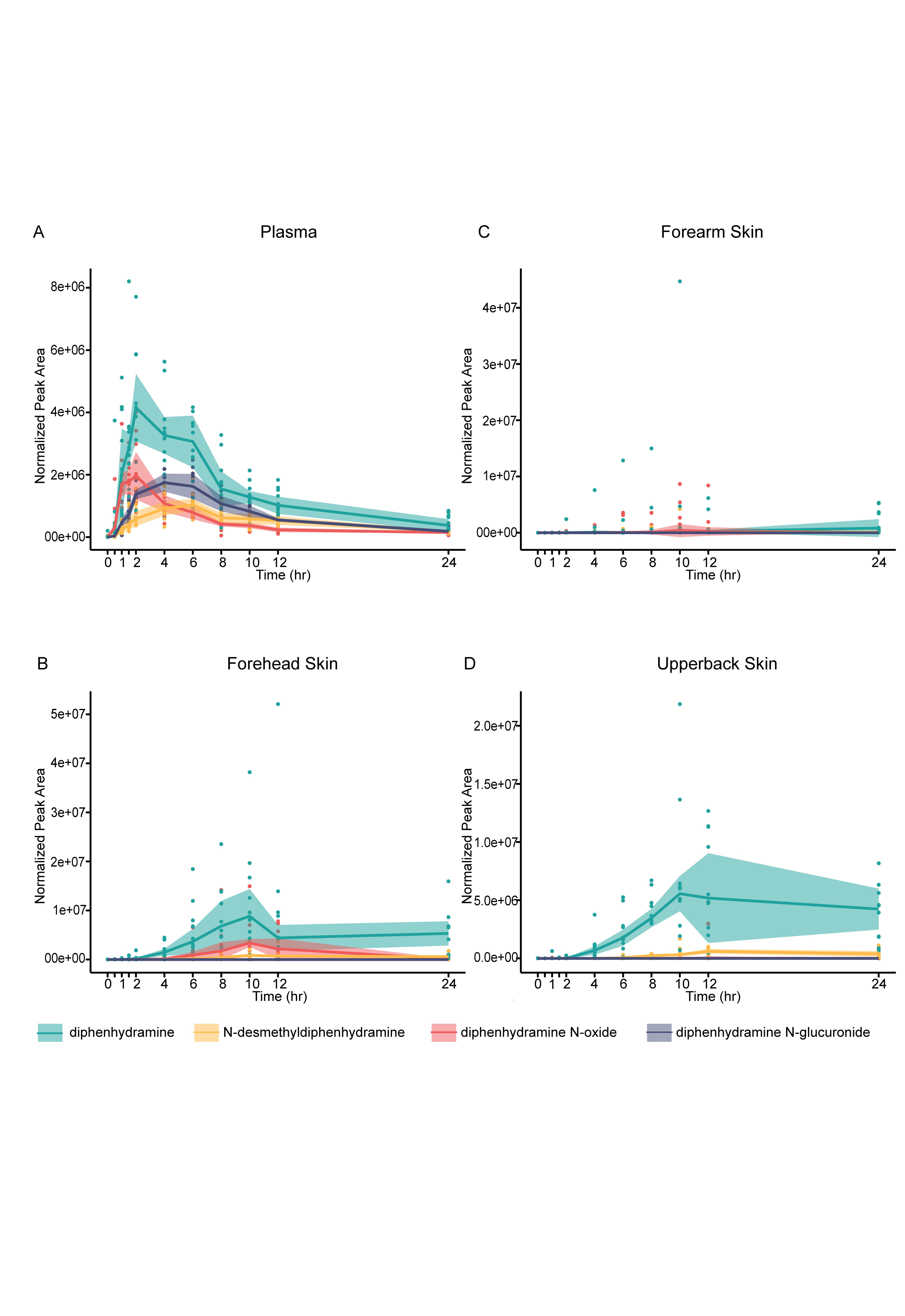

Supplement: S2 Fig — Plot of time vs. peak area for diphenhydramine, N-desmethyldiphenhydramine, diphenhydramine N-oxide and diphenhydramine N-glucuronide in (A) plasma, (B) forehead skin, (C) forearm skin, and (D) upper back skin. The highlighted portion of these plots represent the interquartile range for each sample type and the solid line represents the median values. (TIF) [file pone.0271794.s003.tif]

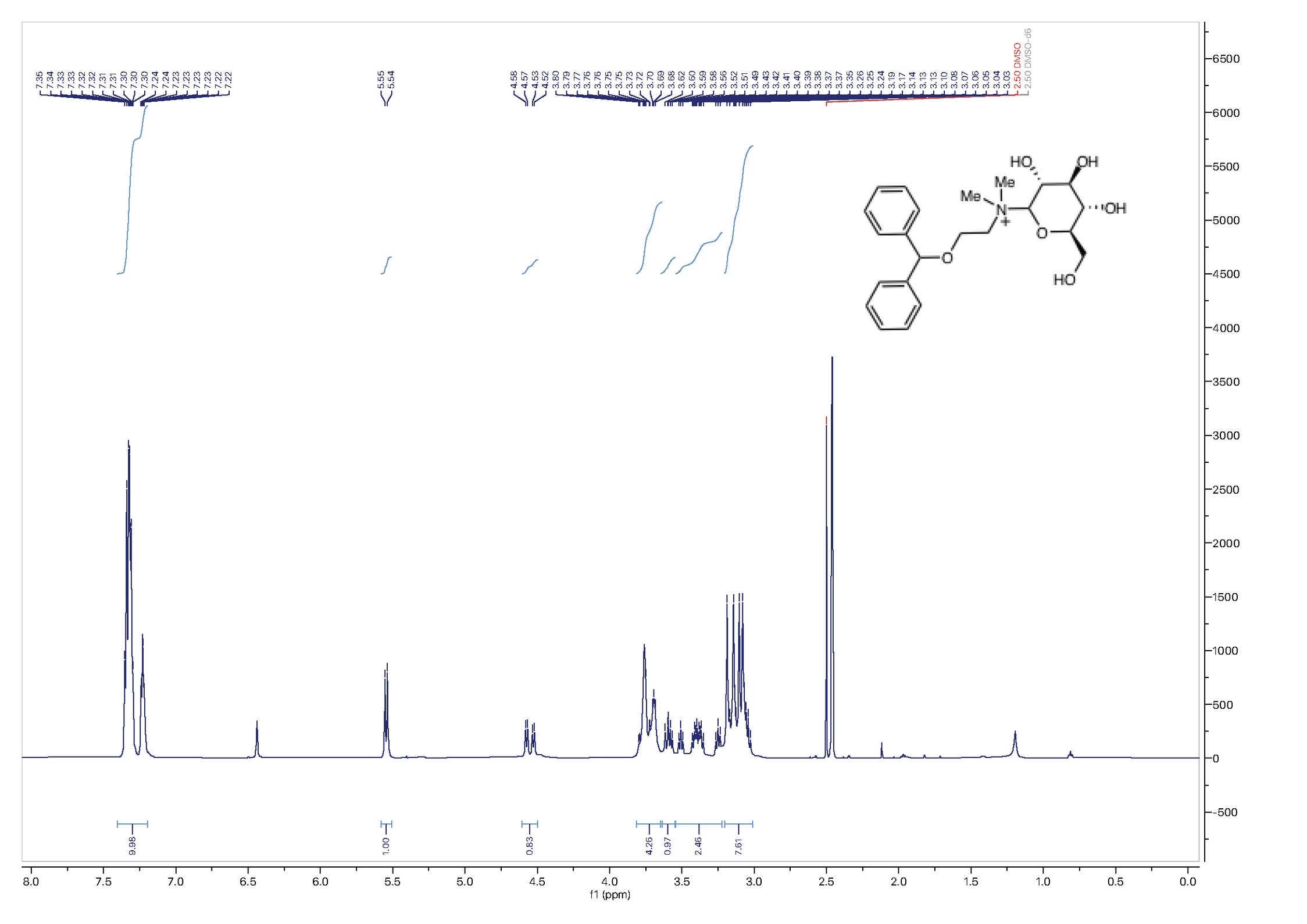

Supplement: S3 Fig — (TIF) [file pone.0271794.s004.tif]

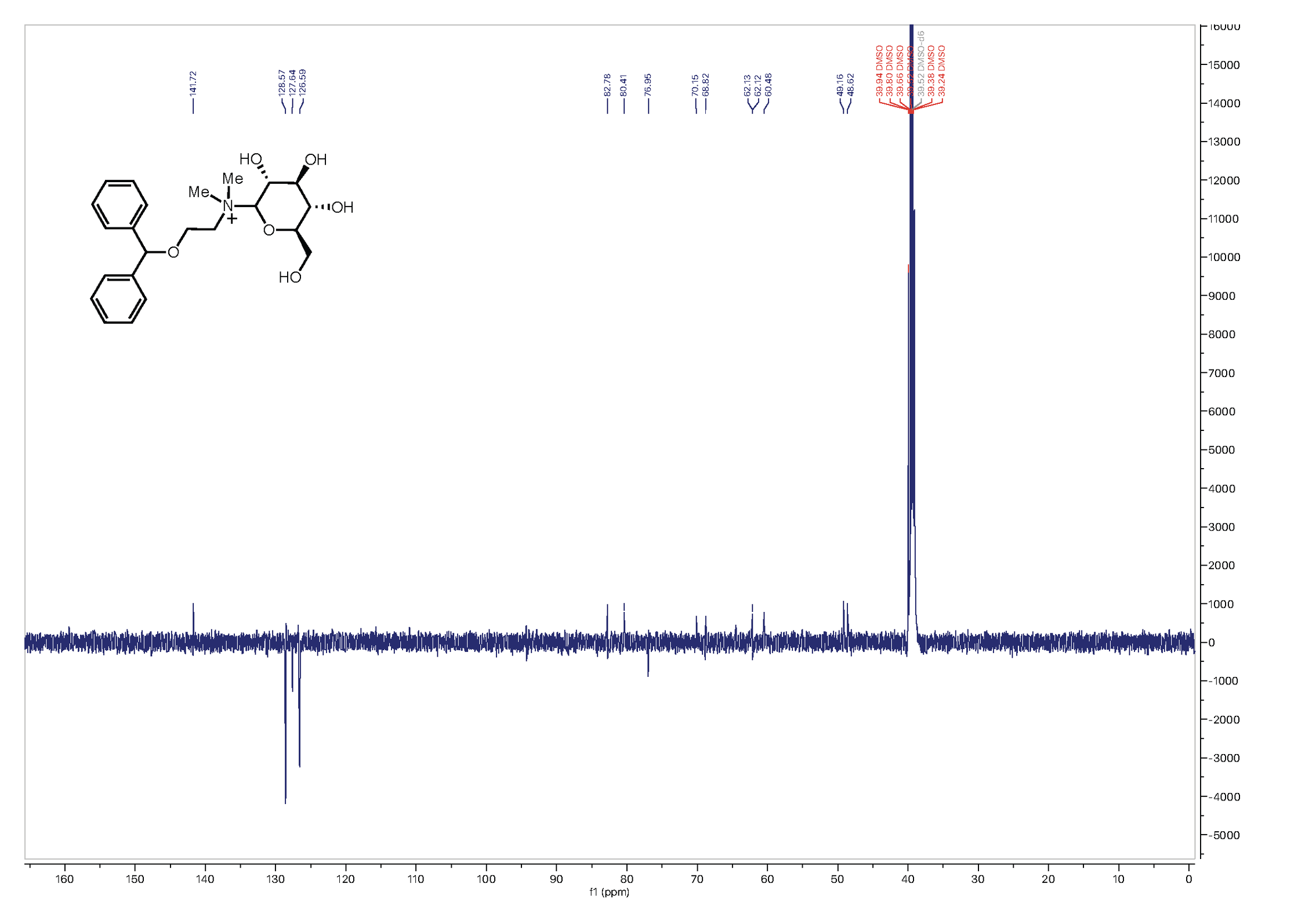

Supplement: S4 Fig — (TIF) [file pone.0271794.s005.tif]
