# Supplementary material for: Non-invasive skin sampling detects systemically administered drugs in humans
Source: PLoS One. 2022 Jul 26;17(7):e0271794. doi: 10.1371/journal.pone.0271794 (PMC9321436; doi:10.1371/journal.pone.0271794)
Supplement: Attachment — Submitted filename: Diphen Manuscript PLOSOne ResponsetoReviewers.docx [file pone.0271794.s006.docx]

When submitting your revision, we need you to address these additional requirements.

1. Please ensure that your manuscript meets PLOS ONE's style requirements, including those for file naming. The PLOS ONE style templates can be found at

[https://journals.plos.org/plosone/s/file?id=wjVg/PLOSOne_formatting_sample_main_body.pdf](https://urldefense.com/v3/__https:/journals.plos.org/plosone/s/file?id=wjVg*PLOSOne_formatting_sample_main_body.pdf__;Lw!!LLK065n_VXAQ!znMPDFOg_j0nlGknp6qUOsrHTDaD7r3dw9dKqUDEEka4R864iS8v_Z7TMLj-MCCejLg$) and

[https://journals.plos.org/plosone/s/file?id=ba62/PLOSOne_formatting_sample_title_authors_affiliations.pdf](https://urldefense.com/v3/__https:/journals.plos.org/plosone/s/file?id=ba62*PLOSOne_formatting_sample_title_authors_affiliations.pdf__;Lw!!LLK065n_VXAQ!znMPDFOg_j0nlGknp6qUOsrHTDaD7r3dw9dKqUDEEka4R864iS8v_Z7TMLj-YY1bt64$)

We have reviewed the style requirements and believe we have now adhered to your requirements.

2. In your Data Availability statement, you have not specified where the minimal data set underlying the results described in your manuscript can be found. PLOS defines a study's minimal data set as the underlying data used to reach the conclusions drawn in the manuscript and any additional data required to replicate the reported study findings in their entirety. All PLOS journals require that the minimal data set be made fully available. For more information about our data policy, please see [http://journals.plos.org/plosone/s/data-availability](https://urldefense.com/v3/__http:/journals.plos.org/plosone/s/data-availability__;!!LLK065n_VXAQ!znMPDFOg_j0nlGknp6qUOsrHTDaD7r3dw9dKqUDEEka4R864iS8v_Z7TMLj-uClBCnM$).

We fully agree with data transparency and have made all of our data available publicly as noted in the following paragraph in the manuscript:

Data Processing and Data Availability

MS data, QExactive files (.raw), were converted to .mzXML files via [MSConvert](http://proteowizard.sourceforge.net/tools.shtml). Feature detection was performed using MZmine2 to produce a feature table containing detected MS1 features with associated peak area, retention time and feature ID number, parameters used can be found in Supplemental Information. All QExactive files (.raw) and .mzXML files, along with method files can be found at MassIVE (https://massive.ucsd.edu/), dataset [MSV000085944](https://massive.ucsd.edu/ProteoSAFe/dataset.jsp?task=beb06102d4064566b1aa943a3ffb1a4d). MS2 fragmentation was analyzed using the GNPS platform. <https://gnps.ucsd.edu/ProteoSAFe/status.jsp?task=deee382b163f4441afea5fda4b2a2bcf>

Upon re-submitting your revised manuscript, please upload your study’s minimal underlying data set as either Supporting Information files or to a stable, public repository and include the relevant URLs, DOIs, or accession numbers within your revised cover letter. For a list of acceptable repositories, please see [http://journals.plos.org/plosone/s/data-availability#loc-recommended-repositories](https://urldefense.com/v3/__http:/journals.plos.org/plosone/s/data-availability*loc-recommended-repositories__;Iw!!LLK065n_VXAQ!znMPDFOg_j0nlGknp6qUOsrHTDaD7r3dw9dKqUDEEka4R864iS8v_Z7TMLj-AltQ7bc$). Any potentially identifying patient information must be fully anonymized.

Important: If there are ethical or legal restrictions to sharing your data publicly, please explain these restrictions in detail. Please see our guidelines for more information on what we consider unacceptable restrictions to publicly sharing data: [http://journals.plos.org/plosone/s/data-availability#loc-unacceptable-data-access-restrictions](https://urldefense.com/v3/__http:/journals.plos.org/plosone/s/data-availability*loc-unacceptable-data-access-restrictions__;Iw!!LLK065n_VXAQ!znMPDFOg_j0nlGknp6qUOsrHTDaD7r3dw9dKqUDEEka4R864iS8v_Z7TMLj-rmrznuU$). Note that it is not acceptable for the authors to be the sole named individuals responsible for ensuring data access.

We will update your Data Availability statement to reflect the information you provide in your cover letter.

3. Thank you for stating the following in the Funding Section of your manuscript:

“This research was partially supported by the National Institutes of Health, Grant UL1TR001442; the Intramural Research Program of National Institute of Environmental Health Sciences of the NIH (ES103363-01, Jarmusch); UCSD Academic Senate Grant (Tsunoda); and NIH R01 GM107550 (Dorrestein).”

We note that you have provided additional information within the Funding Section that is not currently declared in your Funding Statement. Please note that funding information should not appear in the Funding section or other areas of your manuscript. We will only publish funding information present in the Funding Statement section of the online submission form.

Please remove any funding-related text from the manuscript and let us know how you would like to update your Funding Statement. Currently, your Funding Statement reads as follows:

“This research was partially supported by the National Institutes of Health, Grant UL1TR001442; the Intramural Research Program of National Institute of Environmental Health Sciences of the NIH (ES103363-01, AKJ); UCSD Academic Senate Grant (SMT); and NIH R01 GM107550 (PCD)”

Please include your amended statements within your cover letter; we will change the online submission form on your behalf.

4. Thank you for stating the following financial disclosure:

“This research was partially supported by the National Institutes of Health, Grant UL1TR001442; the Intramural Research Program of National Institute of Environmental Health Sciences of the NIH (ES103363-01, AKJ); UCSD Academic Senate Grant (SMT); and NIH R01 GM107550 (PCD)”

Please state what role the funders took in the study. If the funders had no role, please state: "The funders had no role in study design, data collection and analysis, decision to publish, or preparation of the manuscript."

If this statement is not correct you must amend it as needed.

Please include this amended Role of Funder statement in your cover letter; we will change the online submission form on your behalf.

We have placed our amended funding statement including role of funders in the cover letter as you requested.

5. Please include captions for your Supporting Information files at the end of your manuscript, and update any in-text citations to match accordingly. Please see our Supporting Information guidelines for more information: [http://journals.plos.org/plosone/s/supporting-information](https://urldefense.com/v3/__http:/journals.plos.org/plosone/s/supporting-information__;!!LLK065n_VXAQ!znMPDFOg_j0nlGknp6qUOsrHTDaD7r3dw9dKqUDEEka4R864iS8v_Z7TMLj-V9CL0es$).

***We have provided the appropriate captions and matching in-text citations for the SI.***

[Note: HTML markup is below. Please do not edit.]

Reviewers' comments:

Reviewer's Responses to Questions

**Comments to the Author**

1. Is the manuscript technically sound, and do the data support the conclusions?

The manuscript must describe a technically sound piece of scientific research with data that supports the conclusions. Experiments must have been conducted rigorously, with appropriate controls, replication, and sample sizes. The conclusions must be drawn appropriately based on the data presented.

Reviewer #1: Partly

Reviewer #2: Yes

2. Has the statistical analysis been performed appropriately and rigorously?

Reviewer #1: I Don't Know

Reviewer #2: Yes

3. Have the authors made all data underlying the findings in their manuscript fully available?

The [PLOS Data policy](https://urldefense.com/v3/__http:/www.plosone.org/static/policies.action*sharing__;Iw!!LLK065n_VXAQ!znMPDFOg_j0nlGknp6qUOsrHTDaD7r3dw9dKqUDEEka4R864iS8v_Z7TMLj-oCOvAcY$) requires authors to make all data underlying the findings described in their manuscript fully available without restriction, with rare exception (please refer to the Data Availability Statement in the manuscript PDF file). The data should be provided as part of the manuscript or its supporting information, or deposited to a public repository. For example, in addition to summary statistics, the data points behind means, medians and variance measures should be available. If there are restrictions on publicly sharing data—e.g. participant privacy or use of data from a third party—those must be specified.

Reviewer #1: Yes

Reviewer #2: Yes

4. Is the manuscript presented in an intelligible fashion and written in standard English?

PLOS ONE does not copyedit accepted manuscripts, so the language in submitted articles must be clear, correct, and unambiguous. Any typographical or grammatical errors should be corrected at revision, so please note any specific errors here.

Reviewer #1: Yes

Reviewer #2: Yes

5. Review Comments to the Author

Please use the space provided to explain your answers to the questions above. You may also include additional comments for the author, including concerns about dual publication, research ethics, or publication ethics. (Please upload your review as an attachment if it exceeds 20,000 characters)

Reviewer #1: Panitchpakdi et al. report detection of diphenhydramine and its metabolites on skin. They take advantage of a well-established swab sampling method and untargeted mass spectrometric detection. The protocol seems to be detailed sufficiently. The report may be of interest to some other scientists focused on drug metabolism and pharmacokinetics.

Comments:

- How do we know if the amounts of skin excretions collected by skin swabbing are always the same? Moreover, sweating rates may vary among the subjects and over time. Does this variability affect the results?

Thank you for these points that are relevant to the future of this work. Individuals swabbing the skin were instructed to swab for 10 seconds in a circular motion to maintain consistency; however, we are unable to account for varying amounts of skin excretion and sweat variability between individuals. Variability between excretion and sweat may affect these results and likely additional factors such as dehydration and thickness of the skin will require additional studies to optimize analyte detection on skin.

- The mean age of participants (26.3) is rather low. How does it affect the results?

This is an excellent question and according to the literature (1-5 listed below) there can be variation in sweating and skin excretions based on age, this suggests that the results could vary some between age groups. The emphasis of this study was to see if diphenhydramine could be detected on the skin over time, for future studies a larger cohort with a wider age range of participants will ideally help us to understand analyte detection variance between different age groups.

1. Inoue Y, Shibasaki M. Regional differences in age-related decrements of the cutaneous vascular and sweating responses to passive heating. Eur J Appl Physiol Occup Physiol. 1996;74(1-2):78-84. doi: 10.1007/BF00376498. PMID: 8891504.
2. Inoue Y, Nakao M, Araki T, Murakami H. Regional differences in the sweating responses of older and younger men. J Appl Physiol (1985). 1991 Dec;71(6):2453-9. doi: 10.1152/jappl.1991.71.6.2453. PMID: 1778946.
3. Foster KG, Ellis FP, Doré C, Exton-Smith AN, Weiner JS. Sweat responses in the aged. Age Ageing. 1976 May;5(2):91-101. doi: 10.1093/ageing/5.2.91. PMID: 1274803.
4. Ezure T, Amano S, Matsuzaki K. Aging-related shift of eccrine sweat glands toward the skin surface due to tangling and rotation of the secretory ducts revealed by digital 3D skin reconstruction. Skin Res Technol. 2021 Jul;27(4):569-575. doi: 10.1111/srt.12985. Epub 2021 Feb 12. PMID: 33576542; PMCID: PMC8359204.
5. Smith CJ, Alexander LM, Kenney WL. Nonuniform, age-related decrements in regional sweating and skin blood flow. Am J Physiol Regul Integr Comp Physiol. 2013 Oct 15;305(8):R877-85. doi: 10.1152/ajpregu.00290.2013. Epub 2013 Aug 7. PMID: 23926135; PMCID: PMC3798768.

- Can the peak areas in Figure 1 be converted to concentrations of these analytes in sweat?

Provided that sweat volume was not accounted for in this study the peak areas represented in Figure 1 are only able to be representative of relative abundance. Obtaining concentrations and fine tuning the sampling method for detecting and quantitating analytes on the skin is the intended direction of future studies.

- The report does not mention some relevant previous reports and reviews, e.g. J. Mass Spectrom. 2015, 50, 1321; JALM 2020, 5, 877; Trends Endocrinol. Metab. 2021, 32, 66.

Thank you for these relevant references, J. Mass Spectrom. 2015, 50, 1321 and JALM 2020, 5, 877 are now mentioned in the introduction of the manuscript. As shown here “While some forms of skin monitoring exist, for example, sweat testing for cystic fibrosis, ([2](https://paperpile.com/c/IZy0XR/GX9f)) sweat patches such as PharmCheck[(3)](https://paperpile.com/c/IZy0XR/vE2a) and hydrogel micro patches ([~~4~~](https://paperpile.com/c/IZy0XR/CugM),[4](https://analyticalsciencejournals.onlinelibrary.wiley.com/doi/full/10.1002/jms.3702)-[6](https://doi.org/10.1021/ac4039338)) have been utilized, but these are specific to testing topical, hydrophilic substances secreted with sweat.” See line 72 page 2

- Reference 4 is a review on hydrogel-based devices for biomedical applications. Based on its content, it is not justified to cite it after the words “and hydrogel micro patches”. Only reference 5 (or other relevant papers) should be cited in this place.

Reference 4 “Deligkaris, K. et al. 2010. Hydrogel-Based Devices for Biomedical Applications. Sensors and Actuators. B, Chemical 147 (2): 765–74.” has been removed from the manuscript.

- The authors point out the limitations of the other skin sampling methods (“but these are limited to testing topical, hydrophilic substances secreted with sweat”). There is no evidence that the sampling method used in this study performs better because there is no systematic comparison.

Excellent point, many skin sampling methods are very novel and therefore comparisons between our methodology and other sampling methods that specifically analyze sweat are challenging. The referencing in the particular sentence mentioned above has been modified to reflect that skin sampling methods exist but are different from the methodology we have used in our study.

- “co-eluting ion of m/z 418.222”: Is this species in ionic form already in the column? Or, does it become ion in the ion source, i.e. after being eluted from the column?

We synthesized the ion in pure form with m/z of 418.222 and that co-eluted with our samples, indicating that it was in the ionic form when it was in the column. If this species were an in-source fragment it would not have the same retention time as the pure synthesized standard.

- Some of the text/numbers in Figure 2 are too small to read. This figure has been updated to improve legibility.

- Some lines in Figure 2 are so thin that they are barely visible. This figure has been updated to improve legibility.

Reviewer #2: This article describes the use of liquid chromatography coupled to tandem mass spectrometry (LC-MS/MS) for the analysis of swabbed skin samples to detect orally administered diphenhydramine and its metabolites. The article is brief, but well written and includes a nicely designed study combining human plasma measurements as well as non-invasive skin sampling. I only have minor comments and questions that I hope will be of use:

Specific Comments:

1. What is the clinical relevance/usage of diphenhydramine?

Diphenhydramine is a widely-used over-the-counter antihistamine medication utilized for allergies and hypersensitivity conditions. We utilized diphenhydramine in this study because we had seen it before in previous skin studies, it is non-toxic, and widely available.

2. References to studies examining exogenous compounds from latent fingerprints are relevant to the current work and should be cited, including:

Thank you for these references. These fit within the context of our introduction section and have been added in as follows “Additional studies have demonstrated individuals fingerprints as a site of detection for a wide range of chemical compounds.(13-19)” See line 92 page 2, along with one additional reference

17. Ismail M, Costa C, Longman K, Chambers MA, Menzies S, Bailey MJ. Potential to Use Fingerprints for Monitoring Therapeutic Levels of Isoniazid and Treatment Adherence. ACS Omega [Internet]. 2022 Apr 21; Available from: <https://doi.org/10.1021/acsomega.2c01257> that was recently published after our first submission (line 387 page 9 - reference 17).

a. Hinners, O’Neil, Lee, “Revealing Individual Lifestyles through Mass Spectrometry Imaging of Chemical Compounds in Fingerprints,” Scientific Reports, 2018, 8, 5149.

b. Bailey, et al. “Rapid detection of cocaine, benzoylecgonine and methylecgonine in fingerprints using surface mass spectrometry,” Analyst, 2015, 140, 6254-6259.

c. Guinan, Vedova, Kobus, Voelcker, “Mass spectrometry imaging of fingerprint sweat on nanostructured silicon,” Chemical Communications, 2015, 51, 6088–6091.

d. Lauzon, Dufresne, Chauhan, Chaurand, “Development of laser desorption imaging mass spectrometry methods to investigate the molecular composition of latent fingermarks,” Journal of the American Society of Mass Spectrometry, 2015, 26, 878–886.

e. Groeneveld, de Puit, Bleay, Bradshaw, Francese, “Detection and mapping of illicit drugs and their metabolites in fingermarks by MALDI MS and compatibility with forensic techniques,” Scientific Reports, 2015, 5, 11716.

f. Kaplan-Sandquist, LeBeau, Miller, “Chemical analysis of pharmaceuticals and explosives in fingermarks using matrix-assisted laser desorption ionization/time-of-flight mass spectrometry,” Forensic Science International, 2014, 235, 68–77.

3. The m/z values at which the mass resolving powers (i.e., 35,000 and 17,500) are measured should be reported.

The resolving powers are described in the methods section where MS1 scans were collected at 35,000 resolution and 17,500 resolution for MS2 scans. See line 185 page 4.

“MS^1^ scans were collected at 35,000 resolution from *m/z* 150 to 1500 and were performed (~7 Hz) with a maximum injection time of 100 ms, 1 microscan, and an automatic gain control target of 1x10^6^. The top 5 most abundant precursor ions in the MS^1^ scan were selected for fragmentation with an *m/z* isolation width of 1.5 and subsequently fragmented with stepped normalized collision energy of 20, 30, and 40. The MS^2^ data was collected at 17,500 resolution with a maximum injection time of 100 ms, 1 microscan, and an automatic gain control target of 5x10^5^.”

4. The figures are a bit blurry and the data difficult to read.

Text and resolution of figures have been adjusted to make figures more legible and clear.

5. “Further, spectral evidence (exact mass) indicated…” should read “Further, spectral evidence (accurate mass) indicated…” (i.e., exact mass is theoretical, accurate mass refers to a measured value).

Exact mass is now reflected as accurate mass in the manuscript see line 299 page 7.

“Further, spectral evidence (accurate mass) indicated a molecular formula of C23H32NO6+ and the presence of a covalently bonded hexose sugar (neutral loss in the MS/MS spectrum).”

6. PLOS authors have the option to publish the peer review history of their article ([what does this mean?](https://urldefense.com/v3/__https:/journals.plos.org/plosone/s/editorial-and-peer-review-process*loc-peer-review-history__;Iw!!LLK065n_VXAQ!znMPDFOg_j0nlGknp6qUOsrHTDaD7r3dw9dKqUDEEka4R864iS8v_Z7TMLj-sydyrd8$)). If published, this will include your full peer review and any attached files.

If you choose “no”, your identity will remain anonymous but your review may still be made public.

**Do you want your identity to be public for this peer review?** For information about this choice, including consent withdrawal, please see our [Privacy Policy](https://urldefense.com/v3/__https:/www.plos.org/privacy-policy__;!!LLK065n_VXAQ!znMPDFOg_j0nlGknp6qUOsrHTDaD7r3dw9dKqUDEEka4R864iS8v_Z7TMLj-jgMehd4$).

Reviewer #1: No

Reviewer #2: No
